# Shotgun metagenomics analysis indicates *Bradyrhizobium* spp. as the predominant genera for heavy metal resistance and bioremediation in a long-term heavy metal-contaminated ecosystem

**DOI:** 10.1128/mra.00245-24

**Published:** 2024-11-05

**Authors:** Rohan Agashe, Jonathan George, Ashish Pathak, Olasunkanmi Fasakin, John Seaman, Ashvini Chauhan

**Affiliations:** 1 School of the Environment, Florida A&M University, Tallahassee, Florida, USA; 2 Savannah River Ecology Laboratory, University of Georgia, Aiken, South Carolina, USA; Portland State University, Portland, Oregon, USA

**Keywords:** *Bradyrhizobium*, heavy metal resistance, shotgun metagenomics, bioremediation

## Abstract

Ten soil cores were collected from the long-term heavy metal-contaminated Savannah River Site (SRS) and studied using shotgun metagenomics. In-line with our previous reports, *Bradyrhizobium* spp. dominated the SRS soils, and thus we recommend that SRS bioremediation studies target the *Bradyrhizobium* genus.

## ANNOUNCEMENT

Heavy metal contamination is still pervasive at several locations within the Savannah River System (SRS), a former nuclear material fabrication facility (1). Because the native soil microorganisms can be employed to remediate environmental heavy metals (2–8), 10 sediment cores were collected from the top 6 inches from the SRS Steed Pond area (Table 1), by using a JMC 18′′ sampler. Cores were stored on ice and shipped overnight to FAMU where DNA was immediately extracted using the DNeasy PowerLyzer Kit (QIAGEN) from 250 mg of soil. DNA libraries were prepared for shotgun sequencing using the Illumina Nextera Library Prep Kit (Illumina, San Diego, CA, USA), following the manufacturer’s instructions. Purity of the isolated DNA was evaluated using a spectrophotometer (NanoDrop Technologies, Wilmington, DE, USA) and processed for shotgun sequencing using standard protocols on an Illumina Next seq 500 platform (4, 5). All software were run under default mode, unless stated otherwise. Quality of the obtained sequences was evaluated using FastQC v0.11101 adapters, which were removed using cutadapt v2.6102 (9) and reads trimmed by Trimmomatic v0.36103 (10). Low-quality sequences were detected with Komplexity v0.3.6104 (11) and those less than 100 bp were discarded. Taxonomic assignment on the raw sequence reads was performed using the Sequence Taxonomic Analysis Tool (STAT) (12) via the National Center for Biotechnology Information (NCBI) using Google cloud BigQuery commands [(13); https://doi.org/10.6084/m9.figshare.26304805].

**TABLE 1:** Concentrations of the two major heavy metal contaminants typically found in the contaminated Steed Pond area of the Savannah River System, USA^*a*^

Sediment soil core	GPS coordinates	Uranium (mg/kg)	Nickel (mg/kg)	SRA accession	Sample ID	Raw reads
1	33° 19′ 24″ N 81° 43′ 7.4″ W	6814.041	1611.296	SRX14484175	SAMN26725144	1,090,569
2	33° 19′ 24.3″ N 81° 43′ 6.6″ W	4705.779	1386.948	SRX14484176	SAMN26725145	957,982
3	33° 19′ 24.3″ N 81° 43′ 6.4″ W	2665.854	542.532	SRX14484177	SAMN26725146	721,748
4	33° 19′ 24.5″ N 81° 43′ 5.8″ W	2289.978	745.910	SRX14484178	SAMN26725147	1,072,608
5	33° 19′ 24.8″ N 81° 43′ 5.3″ W	2492.864	616.282	SRX14484179	SAMN26725148	976,984
6	33° 19′ 24.5″ N 81° 43′ 4.9″ W	223.741	78.829	SRX14484180	SAMN26725149	1,021,578
7	33° 19′ 24.9″ N 81° 43′ 5.2″ W	1308.968	392.673	SRX14484181	SAMN26725150	827,733
8	33° 19′ 24.1″ N 81° 43′ 5.3″ W	1517.776	354.815	SRX14484182	SAMN26725151	770,826
9	33° 19′ 23.5″ N 81° 43′ 5.6″ W	10439.160	2275.324	SRX14484183	SAMN26725152	1,162,629
10	33° 19′ 24.0″ N 81° 43′ 6.8″ W	2313.841	833.388	SRX14484184	SAMN26725153	934,495

^*a*^ The 10 SRA files can be accessed directly at SRP364474.

Soil heavy metal concentrations were analyzed according to EPA Method 6020A (USEPA, 2007; Method 6020A, Rev. 1). Uranium and nickel concentrations were determined by acid digestion followed by inductively coupled plasma-mass spectrometry on a NexIon 300 (Perkin Elmer, Inc., Waltham, MA, USA) using EPA method 6020B (USEPA, 2014; Method 6020B, Rev. 2).

**Fig 1:**
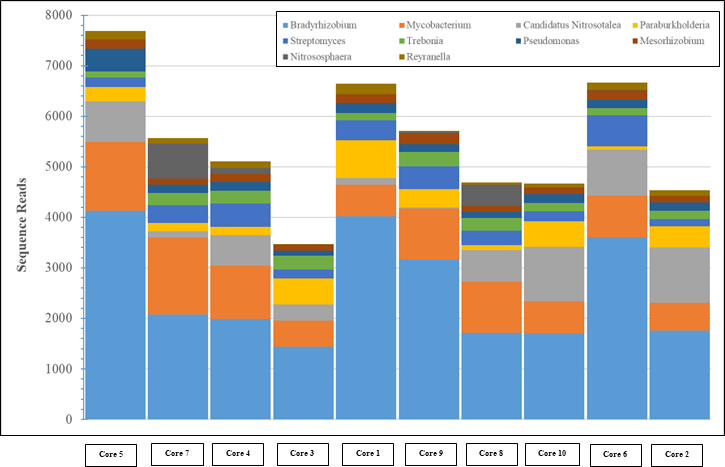
Shown is a stacked bar plot of the top 10 bacterial genera identified using NCBI’s STAT tool in soil cores collected from the long-term heavy metal-contaminated Steed Pond area, Savannah River System, USA.

Among the bacterial sequences identified in the cores, *Bradyrhizobium* spp. dominated (Fig. 1). Interestingly the cores 9, 1, and 2 having the highest U and Ni concentrations showed higher number of sequences belonging to *Bradyrhizobium* spp., occurring at 33%, 38% and 25%, respectively. *Bradyrhizobium* species have not only been identified as a heavy metal-resistant bacteria in other reports (14, 15) but also in our previous SRS studies (3–6). Thus, it appears that *Bradyrhizobium* genus is likely a core bacterial group found in heavy metal-contaminated soils. It is likely that the heavy metal resistance and remediation by *Bradyrhizobium* are facilitated by the presence of key traits, such as the presence of multiple efflux pumps and membrane transporters (2, 5), which collectively facilitates colonization of this genus in long-term contaminated SRS soils. Overall, this study suggests that further studies using *Bradyrhizobium* spp. isolated from the SRS soils will likely pave the way for precise bioremediation, restoration, and management of the long-term contaminated SRS ecosystem.

## Data Availability

The metagenome sequences obtained from this research have been deposited in NCBI under the Sequence Read Archive with BioProject number PRJNA816857. Also, the SRA files are linked in Table 1.
